# The *Aspergillus flavus* Homeobox Gene, *hbx1*, Is Required for Development and Aflatoxin Production

**DOI:** 10.3390/toxins9100315

**Published:** 2017-10-12

**Authors:** Jeffrey W. Cary, Pamela Harris-Coward, Leslie Scharfenstein, Brian M. Mack, Perng-Kuang Chang, Qijian Wei, Matthew Lebar, Carol Carter-Wientjes, Rajtilak Majumdar, Chandrani Mitra, Sourav Banerjee, Anindya Chanda

**Affiliations:** 1 Food and Feed Safety Research Unit, USDA/ARS, Southern Regional Research Center, New Orleans, LA 70124, USA; pamyvette@cox.net (P.H.-C.); les.scharfenstein@ars.usda.gov (L.S.); brian.mack@ars.usda.gov (B.M.M.); perngkuang.chang@ars.usda.gov (P.-K.C.); qijian.wei@ars.usda.gov (Q.W.); matthew.lebar@ars.usda.gov (M.L.); carol.carter@ars.usda.gov (C.C.-W.); raj.majumdar@ars.usda.gov (R.M.); 2 Environmental Health Sciences, Arnold School of Public Health, University of South Carolina, Columbia, SC 29208, USA; cmitra@email.sc.edu (C.M.); achanda@mailbox.sc.edu (A.C.); 3 Department of Mechanical Engineering, University of South Carolina, Columbia, SC 29208, USA; banerjes@cec.sc.edu

**Keywords:** homeobox, *Aspergillus flavus*, development, secondary metabolism, aflatoxin

## Abstract

Homeobox proteins, a class of well conserved transcription factors, regulate the expression of targeted genes, especially those involved in development. In filamentous fungi, homeobox genes are required for normal conidiogenesis and fruiting body formation. In the present study, we identified eight homeobox (*hbx*) genes in the aflatoxin-producing ascomycete, *Aspergillus flavus*, and determined their respective role in growth, conidiation and sclerotial production. Disruption of seven of the eight genes had little to no effect on fungal growth and development. However, disruption of the homeobox gene AFLA_069100, designated as *hbx1*, in two morphologically different *A. flavus* strains, CA14 and AF70, resulted in complete loss of production of conidia and sclerotia as well as aflatoxins B_1_ and B_2_, cyclopiazonic acid and aflatrem. Microscopic examination showed that the Δ*hbx1* mutants did not produce conidiophores. The inability of Δ*hbx1* mutants to produce conidia was related to downregulation of *brlA* (bristle) and *abaA* (abacus), regulatory genes for conidiophore development. These mutants also had significant downregulation of the aflatoxin pathway biosynthetic genes *aflC*, *aflD*, *aflM* and the cluster-specific regulatory gene, *aflR*. Our results demonstrate that *hbx1* not only plays a significant role in controlling *A. flavus* development but is also critical for the production of secondary metabolites, such as aflatoxins.

## 1. Introduction

The filamentous fungus *Aspergillus flavus* produces a number of secondary metabolites including the family of toxic and carcinogenic aflatoxins (AFs). AFs are polyketide-derived compounds that frequently contaminate oil-rich crops such as corn, peanuts, cottonseed, and tree nuts [1]. Ingestion of foods contaminated with AFs has been implicated in acute toxicoses while chronic, low-level exposure can lead to immune suppression and liver cancer [2,3]. In addition to these health risks, there are also significant adverse economic impacts to producers due to market rejection of contaminated crops and livestock losses as well as costs associated with monitoring for AF contamination [4,5].

*Aspergillus flavus* occurs as a saprophyte in soils and normally reproduces clonally by means of conidia (asexual spores). However, a sexual state has been identified in the field indicating that sexual recombination is possible in *A. flavus* [6,7]. Infestations of *A. flavus* in crops are sustained by production and mass dissemination of air-borne conidia or the persistence of sclerotia (aggregates of mycelia that are capable of resisting unfavorable environmental conditions) in soils and on plant debris. The sexual ascospores are found within ascocarps present in the matrix of sclerotia (termed stromata) [7]. Sclerotia can remain dormant for long periods of time until favorable conditions allow germination and production of more conidial inoculum [8,9]. High concentrations of AFs may occur in both conidia and sclerotia of *A. flavus* [10]. Increased animal toxicity has been attributed to the combined activity of AFs and other metabolites present in sclerotia [11,12].

Fungal development and secondary metabolite production are co-regulated [13,14,15]. Environmental factors that affect fungal growth, such as nutritional status, pH, temperature, stress and light are also involved in regulation of developmental processes including sexual or asexual reproduction [8,16,17,18]. Several regulatory factors that govern morphological differentiation in fungi have been identified, especially in *Aspergillus nidulans*, and some of them control genes involved in secondary metabolite production as well [19,20,21,22]. For example, the Velvet complex proteins, LaeA, VeA and VelB, regulate *A. nidulans* development as well as secondary metabolite production in a light-responsive manner [15,23,24,25]. The VeA-LaeA-VelB heterotrimeric complex also appears to have a similar function in *A. flavus* because inactivation of either VeA or LaeA results in loss of AF and sclerotial production [26,27,28,29,30]. The transcription factors, NsdC and NsdD, also regulate both fungal development and secondary metabolism in *A. flavus*, and disruption of respective genes leads to loss of sclerotial and aflatoxin production [31,32].

Homeobox proteins are a class of transcription factors that serve as master regulators of development in many eukaryotes including animals, plants and fungi (reviewed in [33]). The homeodomain (HD) typically consists of about 60 amino acids and is capable of recognizing and binding to specific DNA sequences in the promoters of targeted genes under its control. In animals, HD proteins are encoded by a large number of homeobox genes (up to 200 in humans) that specify body plan while in plants (up to 100 in *Arabidopsis*) they participate in developmental programs of flowers, leaves and vegetative shoots [34,35]. Typically, fungi harbor 6–12 homeobox genes [36]. Studies in fungi have shown that homeobox genes are required for normal conidiogenesis, fruiting body formation, reproduction and virulence. The first described homeobox gene in a filamentous ascomycete was *pah1* of *Podospora anserina*; it regulates production of microconidia and hyphal branching [37]. A systematic deletion of seven HD transcription factors in *P. anserina* has shown that some play a role in shaping of the fruiting body [38]. In a similar study, eight HD transcription factors were knocked out in *Magnaporthe oryzae*, and a number of them are required for proper hyphal growth, conidiation and appressorium development [39,40]. Of the nine homeobox genes predicted in the phytopathogen, *Botrytis cinerea*, the BcHOX8 gene is involved in regulating vegetative growth, conidiation, and the ability to efficiently colonize different host plants [36]. Inactivation of the *htf1* homeobox gene in three species of *Fusarium* results in production of aberrant conidiophore phialides and severe reduction in conidiation [41]. Deletion mutants of the *GRF10* homeobox gene in the human fungal pathogen, *Candida albicans*, also display decreased hyphal growth, defective chlamydospore, biofilm production and attenuated virulence in a mouse model [42].

Homeobox genes have yet to be functionally characterized in the genus *Aspergillus* that includes human, animal and plant pathogens, with the exception of the role of an *Aspergillus fumigatus* gene in calcineurin-mediated stress response [43]. In the present study, we performed a systematic deletion of eight predicted homeobox genes from *A. flavus*. We found that *hbx1* was required for normal vegetative growth and production of conidia and sclerotia as well as the production of secondary metabolites, including aflatoxins.

## 2. Results

### 2.1. Identification and Phylogenetic Analysis of Homeobox Genes of A. flavus

Putative *A. flavus* homeobox genes were identified by querying the NCBI protein database using the term “*Aspergillus flavus* homeobox” and “InterPro homeodomain term (IPR001356)”. The search identified eight genes coding for homeodomain (HD) proteins in the genome of *A. flavus* NRRL3357. These genes were denoted as *hbx* 1–8 (homeobox). A close examination of the domains of these proteins showed that all but *hbx8* (AFLA_114960) had a conserved HD. A comparison of the *A. flavus* NRRL3357 genome sequence to that of *A. flavus* 70 (AF70) and *A. oryzae* RIB40 sequence identified an incorrectly called intron in the *hbx8* gene sequence that resulted in the HD region being truncated. The comparison also identified an additional 65 N-terminal amino acids that were not annotated in the NRRL3357 *Hbx8*. All deduced HD proteins contained the characteristic three alpha helical regions involved in DNA binding. Hbx1, 4, 7 and 8 also contained a short beta-sheet region between the second and third alpha helical regions (Figure S1).

**Figure 1:**
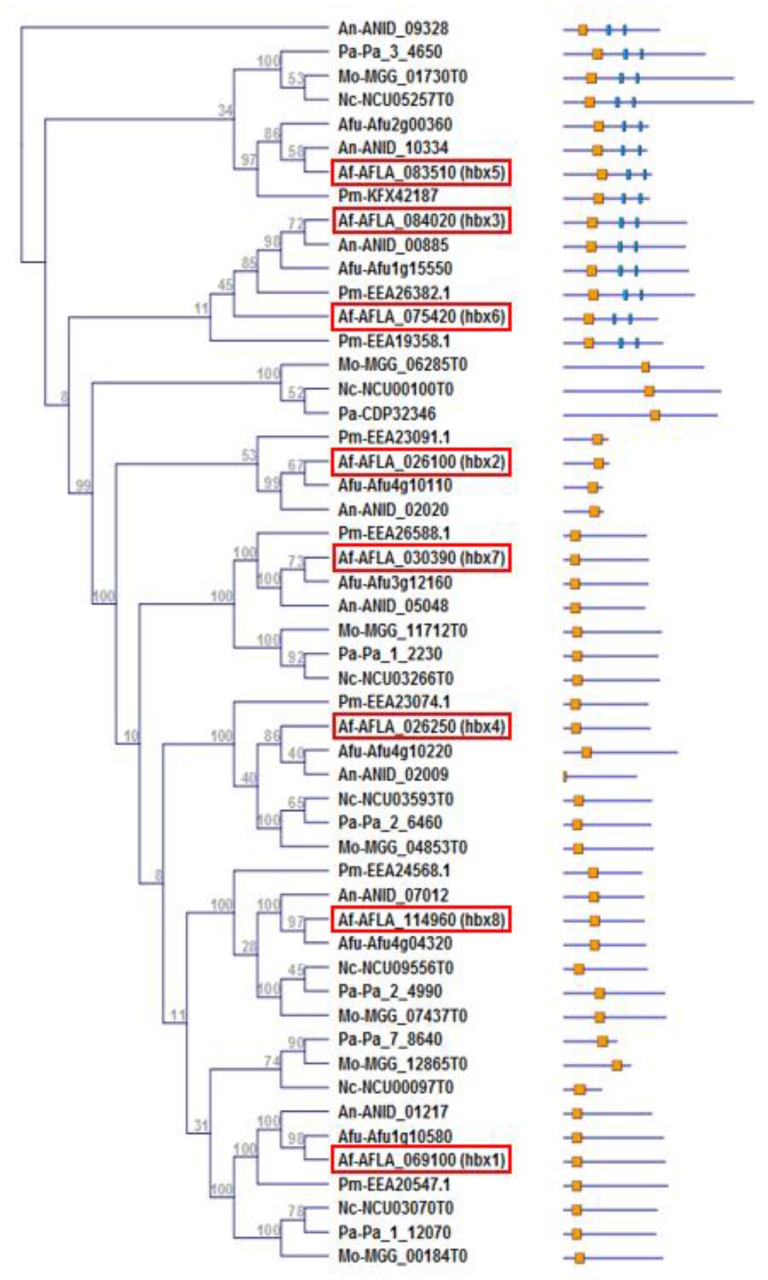
**Phylogenetic analysis of putative homoebox transcription factors**. A neighbor-joining tree was constructed using CLC Sequence Viewer software based on the deduced amino acid sequences of representative fungal homeodomain transcription factors (TFs) identified using the InterPro homeodomain term (IPR001356). Putative *A. flavus* homeobox TF NCBI accession numbers are highlighted in boxes. Domain architecture schematics depict putative homeodomain motifs as orange boxes and Cys(2)-His(2) zinc finger motifs as blue boxes. Abbreviations for fungal species followed by their accession numbers are as follows: AFLA, *Aspergillus flavus*; ANID, *Aspergillus nidulans*; Afu, *Aspergillus fumigatus*; EEA, *Penicillium marneffei*; Pa, *Podospora anserina*; MGG, *Magnaportha oryzae*; NCU, *Neurospora crassa*.

Phylogenetic analysis showed that the eight Hbx proteins were separated into 7 distinct clades with Hbx3 and Hbx6 residing within the same clade (Figure 1). Hbx3, 5 and 6 all contain two C2H2 zinc-finger DNA binding domains (IPR007087) in addition to the HD (IPR001356). Alignment of the deduced amino acid sequences of the *Afhbx* genes indicated that five of the eight *hbx* genes (2, 3, 5, 6 and 8) constituted a class of TALE (Three Amino Acid Loop Extension) genes that contain three additional amino acids between alpha helices 1 and 2 of the homeodomain (Figure S1). Orthologs of each of the eight *Afhbx* genes were identified in other aspergilli such as *A. fumigatus* and *A. nidulans* as well as the related Eurotiomycete, *Penicillium marneffei* (teleomorph-*Talaromyces marneffei*) (Figure 1). Six *Afhbx* orthologs were found to be present in the genomes of the Sordariomycetes, *Neurospora crassa*, *Podospora anserina* and *M. oryzae*; each contained seven homeobox genes. No *Afhbx* ortholog was identified for *P. anserina* (Pa CDP32346; *pah7*) and *N. crassa* (NCU00100), both orthologs of the *M. oryzae* homeobox gene HOX4 (MGG_06285). This class of homeobox proteins contains a characteristic rhodanese homology domain (pfam 00581) and was shown to play a role in determining conidia size in *M. oryzae* [40] and perithecium distribution in *P. anserina* [38].

### 2.2. Phenotypic Analysis of A. flavus hbx (Afhbx) Deletion Mutants

**Figure 2:**
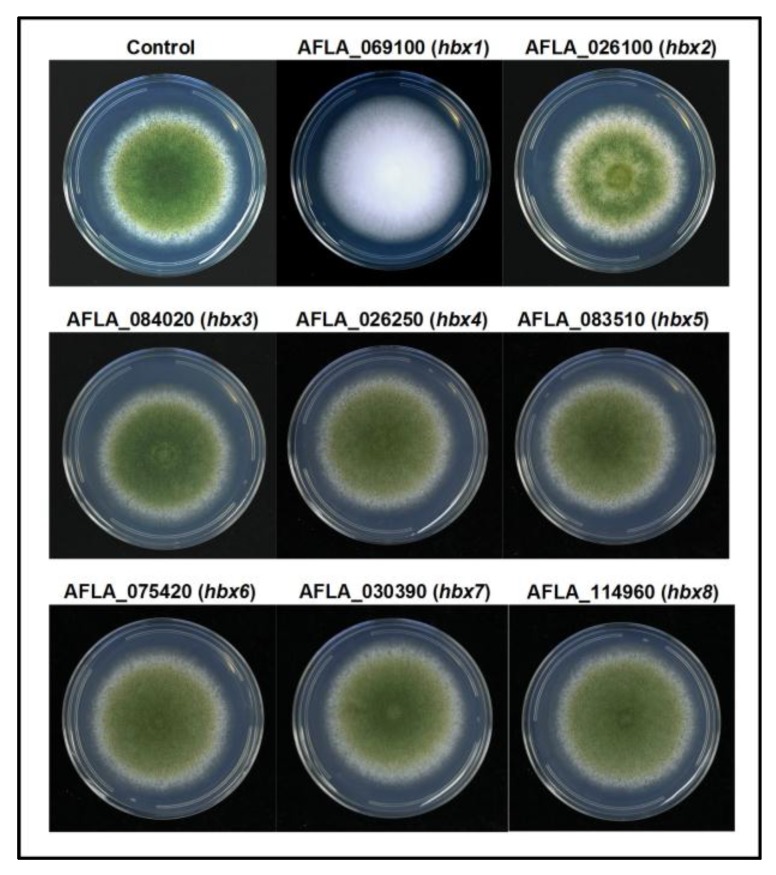
**Colony morphology of *A. flavus* homeobox (*hbx*) deletion mutants.** All strains were grown for 7 d under white light on PDAU at 30 °C. Note loss of conidial production in the CA14 Δ*hbx1* deletion mutant.

**Figure 3:**
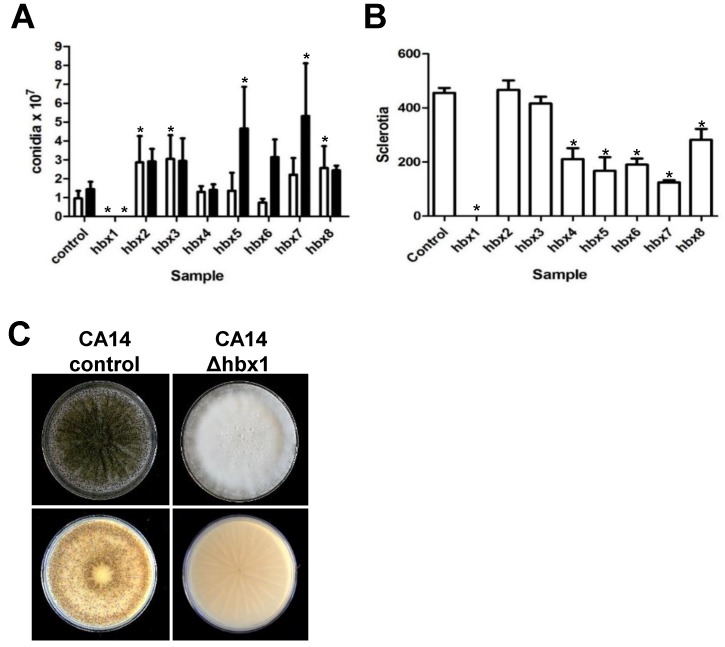
**Analysis of conidial and sclerotial production in CA14 *hbx* mutants.** (**A**) Conidial production in the CA14 control and *hbx* mutants was quantified following growth of fungal strains on PDAU (white bars) or WKMU (black bars) under white light at 30 °C for 7 d. (**B**) Sclerotial production in the CA14 control and *hbx* mutants was quantified following growth of fungal strains on WKMU in the dark at 30 °C for 14 d. Experiments were performed with three biological replicates (with 3 technical replicates of each sample). Statistical analysis was carried out using one-way ANOVA followed by Dunnett’s Multiple Comparison Test. Values of bars with asterisks are significantly different by analysis of variance (*p* < 0.05). (**C**) Sclerotial production by CA14 control and Δ*hbx1* mutant following 14 d growth on WKMU agar plates in the dark at 30 °C. Upper panel depicts top of colony while lower panel shows colony bottom. Note lack of sclerotia in the CA14 Δ*hbx1* mutant.

Disruption of each of the eight *Afhbx* genes in CA14 was confirmed by PCR analysis, and one representative knockout strain of each was selected for further analysis (Figure S2). Loss of *hbx* gene expression in the selected knockout was confirmed by RT-qPCR (Figure S3). The effects of the deletion of the *hbx* genes on colony phenotype, conidiation and sclerotial production were determined. Figure 2 shows the colony phenotype of each CA14 Δ*hbx* mutant and the control strain. No significant difference in growth was observed in any of the CA14 Δ*hbx* mutants compared to the control (data not shown). All CA14 Δ*hbx* mutants produced conidia with the exception of *hbx1*, which exhibited a “fluffy” aconidial phenotype. Conidial production in CA14 Δ*hbx* mutants 5, 6 and 7 was observed to be medium-dependent with a greater number of conidia being produced during growth on WKMU medium compared to PDAU (Figure 3A). A significant increase (*p* ≤ 0.05) in conidial production was observed in Δ*hbx* mutants 2, 3 and 8 compared to the control on PDAU and for mutants 5 and 7 on WKMU. Sclerotial production was significantly (*p* ≤ 0.05) decreased in all CA14 Δ*hbx* mutants with the exception of mutants 2 and 3 (Figure 3B). The CA14 Δ*hbx1* mutant failed to produce sclerotia during growth on WKMU agar (Figure 3B,C). The observed total loss of conidial and sclerotial production in the CA14 Δ*hbx1* mutant led us to focus further efforts on analysis of only the *hbx1* homeobox gene.

**Figure 4:**
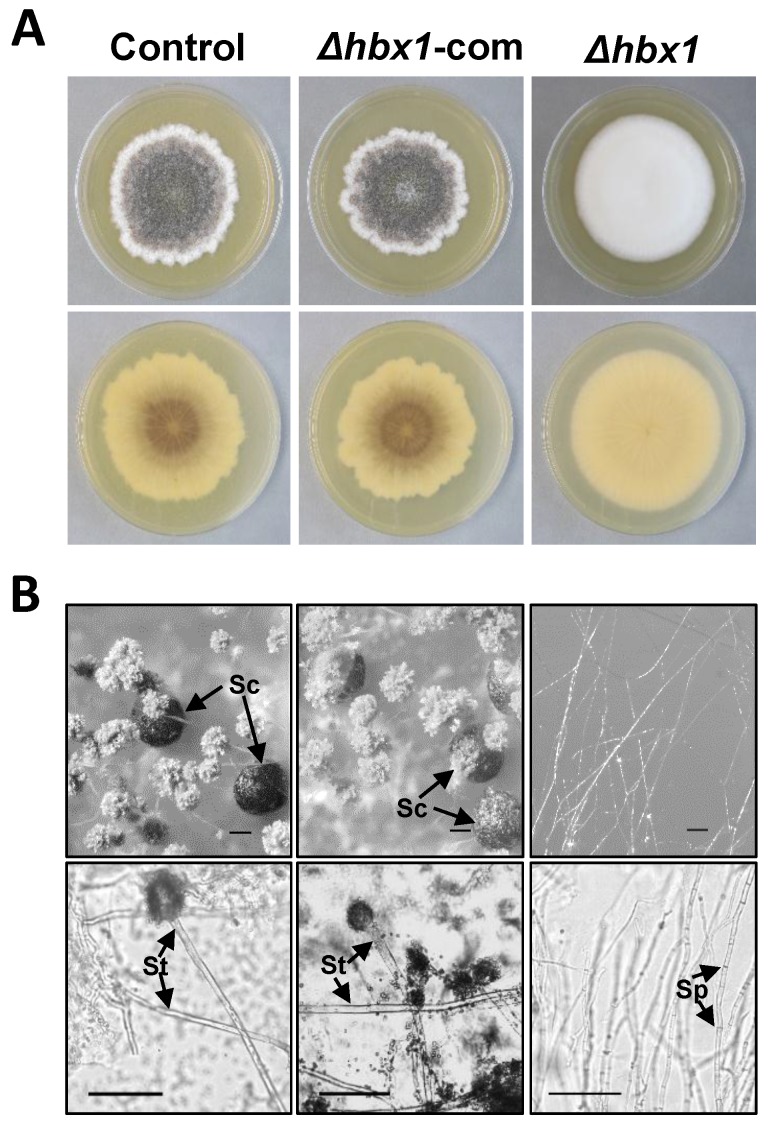
**Examination of conidiophore and sclerotial production in AF70 *hbx* mutants.** (**A**) AF70 pyrG-1 control, Δ*hbx1* #4 mutant and Δ*hbx1*-com #8 genetically complemented strain were grown on WKMU agar for 10 days in the dark at 30 °C. Upper panel depicts top of colony while lower panel shows colony bottom. Note lack of sclerotial and conidiophore production in the Δ*hbx1*mutant. (**B**) Microscopic examination of hyphae, sclerotia and conidiophores. Upper panel: Conidiophore and/or aerial hyphal structures and sclerotia were visualized using a Nikon SMZ25 stereoscope at ×120 magnification. Lower panel: Aerial hyphae were differentiated from conidiophore stipes using a Nikon E600 microscope equipped with a Nikon DS-Qi1Mc camera at ×200 magnification. Note lack of conidiophores and sclerotia in the Δ*hbx1* mutant. Only septate hyphae are present in the mutant. Abbreviations: Sc, sclerotia; St, conidiophore stipe; Sp, hyphal septum. Scale bars, 100 μm.

While CA14 was initially used as host for knock out of all eight of the *A. flavus* homeobox genes, functional characterization of the role of *hbx1* in fungal development and aflatoxin production was performed using strains derived from the AF70 parent as it produced much higher levels of sclerotia and aflatoxins than CA14. Successful disruption of the AF70 *hbx1* homolog was confirmed by PCR in two of three transformants that exhibited lack of conidiation (Figure S4A,B). Loss of *hbx1* expression in the AF70 Δ*hbx1* #4 mutant was confirmed by RT-qPCR (Figure S4C). The AF70 Δ*hbx1* #4 mutant had the similar fluffy colony phenotype observed in the CA14 Δ*hbx1* mutant and also did not produce sclerotia (Figure 4A,B). The AF70 Δ*hbx1* #4 mutant was genetically complemented and a number of positive transformants confirmed by PCR (Figure S5A,B). PCR analysis of three putative AF70 Δ*hbx1*-com transformants confirmed that the wild-type *hbx1* gene and the adjacent *pyrG* selectable marker had integrated into the site of the deleted *hbx1* gene in the mutant (Figure S5B). Gene expression analysis of the genetically complemented AF70 Δ*hbx1*-com #8 showed that *hbx1* expression was restored and this isolate was used in further analyses (Figure S4C). The AF70 *hbx1* mutant showed a statistically significant increase in growth in the light (65.5 ± 2.2 mm) and dark (70.0 ± 2.5 mm) compared to the control (light, 34.8 ± 1.2 mm; dark, 44.0 ± 0.9 mm) and the genetically complemented strain (light, 35.1 ± 0.6 mm; dark, 44.1 ± 0.9 mm).

**Figure 5:**
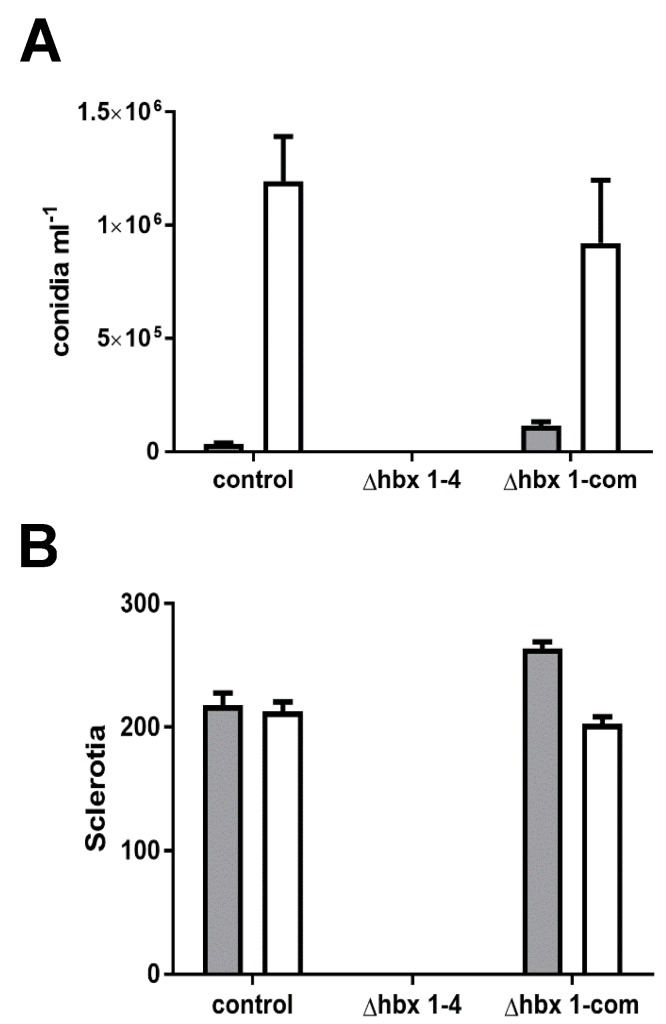
**Conidia and sclerotia production in AF70 strains**. Conidia (**A**) and sclerotia (**B**) production (6 replicates) was quantified after growth for seven (conidia) or 14 days (sclerotia) on WKMU agar under white light or in the dark at 30 °C. White bars: illumination. Dark bars: no illumination.

Microscopic examination of the AF70 Δ*hbx1* mutant failed to detect the presence of conidiophores or sclerotia while both of these structures were observed for the control and the Δ*hbx1*-com #8 strain (Figure 4B). The Δ*hbx1* mutant produced aerial hyphae that were septate and about half the diameter of non-septate conidiophore stipes produced by the control and genetically complemented strain (Figure 4B). Levels of conidia and sclerotia produced by Δ*hbx1*-com #8 were comparable to that produced by the AF70 control strain while the Δ*hbx1* strain failed to produce either conidia or sclerotia under both light and dark growth conditions (Figure 5A,B).

### 2.3. Analysis of hbx1 and Developmental Gene Expression

**Figure 6:**
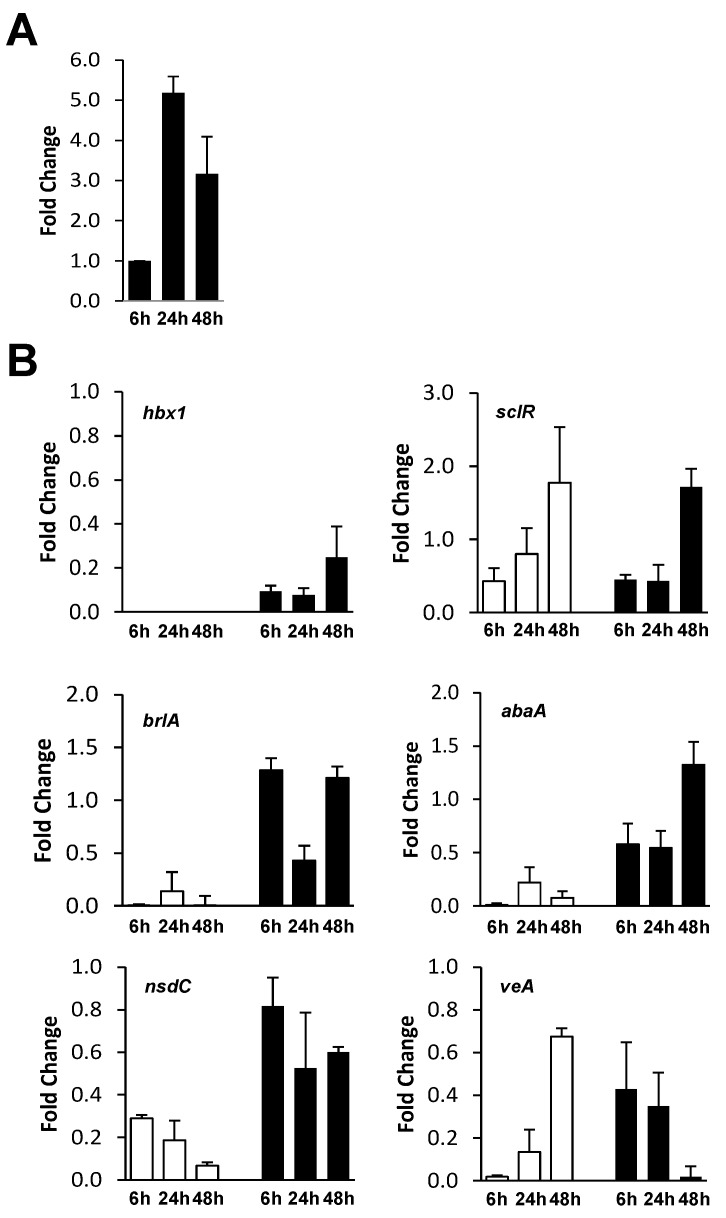
**Analysis of *hbx1* and developmental gene expression in AF70 strains.** (**A**) RT-qPCR of *hbx1* expression in the AF70 pyrG-1 control strain at 6, 24 and 48 h time points. (**B**) Analysis of *hbx1* and developmental gene expression in the AF70 Δ*hbx1* #4 (white bars) and Δ*hbx1*-com #8 strains (black bars). Expression is relative to a level of 1 set for the AF70 control. Cultures were grown and samples prepared as described in Materials and Methods. The relative gene expression levels for all time points were normalized to the *A. flavus* 18S rRNA C_T_ values as part of the ΔΔC_T_ analysis method. A Fold-Change formula (2^−ΔΔC^_T_) [44] was used in the conversion of the ΔΔC_T_ results for a final calculation of values.

Expression of the *hbx1* gene in AF70 was analyzed by RT-qPCR. In the AF70 control, *hbx1* expression increased about 5-fold from 6 to 24 h then decreased by about 40% at 48 h (Figure 6A). As expected, there was no detectable expression of *hbx1* in the AF70 Δ*hbx1* #4 mutant at any of the time points examined and though lower than observed for the control, the Δ*hbx1*-com #8 strain expressed *hbx1* (Figure 6B). The Δ*hbx1*-com #8 strain achieved about 10% of the level of expression of *hbx1* in the control after 6 and 24 h and increased to about 25% of the level of expression of the control at 48 h. Expression of the regulatory genes of conidiophore development, *brlA* and *abaA*, was also analyzed in the AF70 control, Δ*hbx1* and Δ*hbx1*-com strains (Figure 6B). Relative to the Δ*hbx1*-com strain, expression of *brlA* and *abaA* in the AF70 Δ*hbx1* mutant was extremely low at 6 and 48 h and was about 50% of the complemented strain at 24 h (Figure 6B). Expression studies were also performed on *nsdC* and *veA*, encoding global regulators of development and secondary metabolism in *A. flavus*. In general, both *nsdC* and *veA* expression were significantly lower in the Δ*hbx1* mutant compared to the Δ*hbx1*-com strain with the exception of *veA* at 48 h (Figure 6B). Additionally, *sclR*, a regulatory gene of sclerotial morphogenesis in *A. oryzae*, showed no significant difference in expression at any of the time points between the Δ*hbx1* mutant and the Δ*hbx1*-com strain indicating *hbx1* does not play a significant role in regulating expression of *sclR*.

### 2.4. Localization of Hbx1

**Figure 7:**
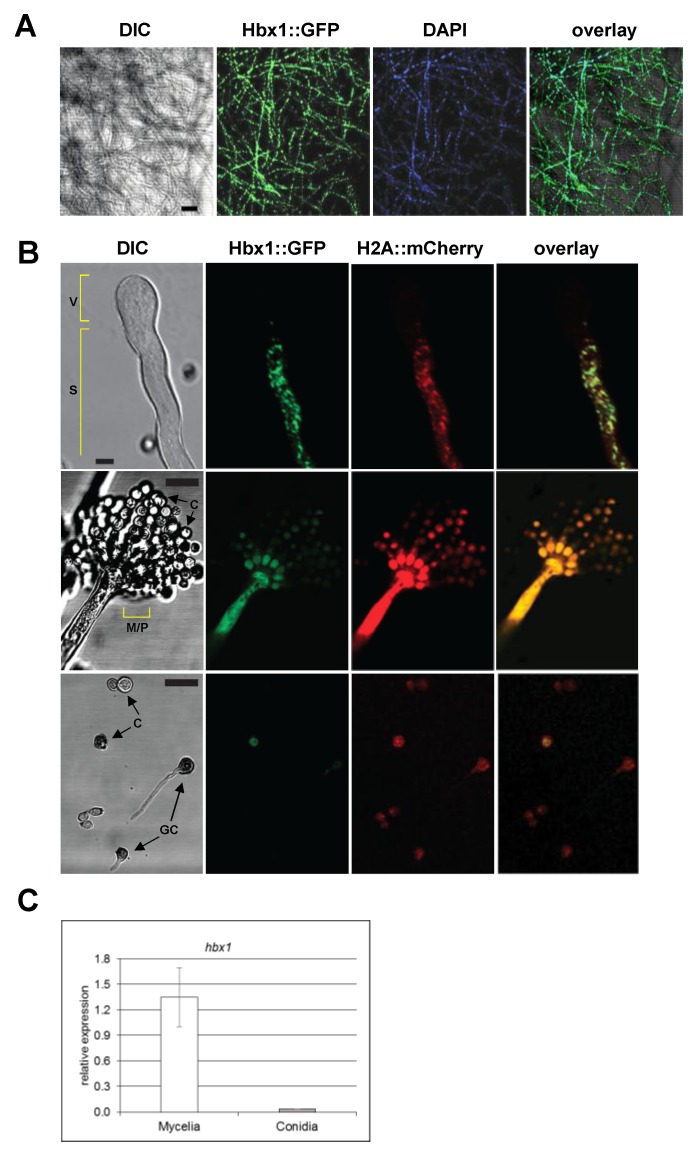
**Localization of Hbx1 protein using CLSM.** (**A**) Analysis of spatial distribution of Hbx1 within mycelia. CA14 expressing Hbx1::GFP was grown on solid PDA growth medium and imaged at 48 h time point. Scale bar, 50 μm. (**B**) Localization of Hbx1 in developing conidiophores. Upper panels: imaging of a conidiophore initiating vesicle formation. CA14 expressing Hbx1::GFP and histone H2A::mCherry was grown on PDA and studied after 48 h. Note lack of Hbx1 and nuclei in the vesicular region. Middle panels: imaging of a mature conidiophore. Note decrease in Hbx1 localization to conidia. Scale Bar, 5 μm; Bottom panels: Conidia (10^3^) of *A. flavus* CA14 expressing Hbx1::GFP and histone H2A::mCherry were inoculated in 10 µL of potato dextrose broth and were observed under CLSM 6 h after inoculation (a time that corresponds to the initiation of spore germination). For both samples, the image acquisition conditions were adjusted such that Hbx-1:GFP could be visualized in at least one conidium. The adjusted image conditions were then propagated to red channel (to visualize H2A::mCherry) as well. Fluorescence intensity was increased in the middle and lower panels in order to clearly see fluorescence in the metulae/phialides and conidiospores. Scale bar, 10 µm. (**C**) RT-qPCR analysis of *hbx1* expression levels in CA14 control grown with agitation in WKMU broth for 2 d (mycelia) or WKMU agar plates for 6 d (isolated conidia). Abbreviations: GC, germinating conidium; C, conidium; M/P, metular/phialide region; V, vesicle; S, stipe.

As shown in Figure 7A, we observed the Hbx1::GFP fusion protein localizing to nuclei in mycelia examined after 48 h growth. Since our microscopic examination of conidiophore development demonstrated that Δ*hbx1* mutants failed to produce conidiophores, a microscopic examination of the spatial distribution of Hbx1 within nuclei of young and mature conidiophores was performed. For this purpose, a dual-color CLSM was performed using the CA14 strain that co-expresses Hbx1::GFP chimera along with histone H2A protein tagged with mCherry. Both Hbx1::GFP and H2A::mCherry co-localized to nuclei that were mostly observed in the stipe region of the young, developing conidiophore that had not yet formed metulae, phialides or conidia (Figure 7B, upper panels). No nuclei were detected in the vesicle at this stage of conidiophore development. In mature conidiophores harboring conidia, the majority of Hbx1::GFP fluorescence was detected in the stipe, vesicular and metular/phialide regions of the conidiophore (Figure 7B, middle panels). The approximately equivalent levels of Hbx1::GFP and H2A::mCherry fluorescence observed in the stipes (Figure 7B, upper panels) compared to lower levels of GFP fluorescence to that of mCherry in 6 h post-inoculated conidia (Figure 7B bottom panels) suggested a lower level of *hbx1* expression in conidia. The presence of lower levels of Hbx1::GFP in conidia compared to mycelia was supported by RT-qPCR analysis of *hbx1* expression that showed significantly higher levels of *hbx1* transcripts in mycelia than in conidia (~2% of mycelial level) (Figure 7C).

### 2.5. Analysis of Aflatoxin Production and Gene Expression in hbx1 Mutants

**Figure 8:**
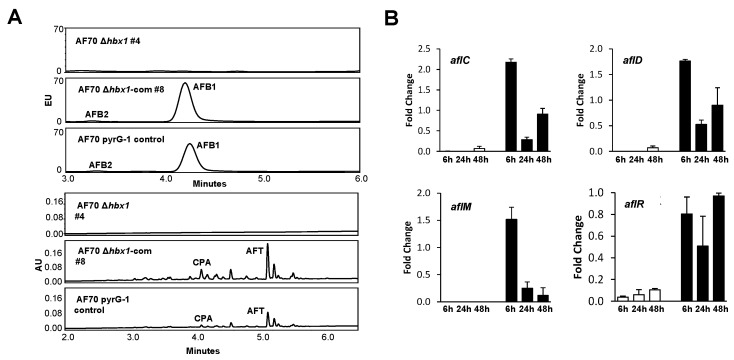
**Analysis of aflatoxin, cyclopiazonic acid and aflatrem production in AF70 strains.** Triplicate samples representing combined lyophilized mycelia and broth were collected following 7 d (aflatoxin) or 14 d (aflatrem and CPA) static growth in WKMU at 30 °C in the light or dark. (**A**) Representative UPLC fluorescence chromatograms of AF70 samples (dark only) extracted and analyzed on a Waters Acquity UPLC system using fluorescence detection (ex = 365 nm, em = 440 nm) for aflatoxin B_1_ (AFB_1_) and B_2_ (AFB_2_) and UPLC-UV detection (280 nm) for cyclopiazonic acid (CPA) and aflatrem (AFT). Standards were used to identify and quantify aflatoxins: AFB_1_ retention time = 4.30 min; AFB_2_, rt = 3.32 min. The approximate limit of detection for AFB_1_ and AFB_2_ was 3 ng/mg sample. CPA, rt = 4.10 min; AFT, rt = 5.19 min. (**B**) RT-qPCR analysis of aflatoxin biosynthetic genes *aflC*, *aflD*, *aflM* and the pathway specific transcriptional activator gene, *aflR*. Cultures of the AF70 Δ*hbx1* #4 (white bars) and Δ*hbx1*-com #8 strains (black bars) were grown as described above for aflatoxin analysis. Expression is relative to a level of 1 set for the AF70 control. The relative gene expression levels for all time points were normalized to the *A. flavus* 18S rRNA C_T_ values as part of the ΔΔC_T_ analysis method. A fold-change formula (2^−ΔΔC^_T_) [44] was used in the conversion of the ΔΔC_T_ results for a final calculation of values.

Aflatoxin analysis by UPLC showed that the AF70 Δ*hbx1* mutant failed to produce AFB_1_ or AFB_2_ (detection limit of ≤3 ng/mg lyophilized sample) while the control strain produced 934.4 ± 356.5 ng/mg AFB_1_/13.8 ± 4.2 ng/mg AFB_2_ in the light and 372.9 ± 44.4 ng/mg AFB_1_/13.9 ± 3.2 ng/mg AFB_2_ in the dark. The genetically complemented strain produced 1408.9 ± 210.8 ng/mg AFB_1_/22.2 ± 14.6 ng/mg AFB_2_ in the light and 472.6 ± 134.6 ng/mg AFB_1_/13.2 ± 3.8 ng/mg AFB_2_ in the dark (Figure 8A and Figure S6). Extracts were also examined for the presence of cyclopiazonic acid (CPA) and aflatrem (AFT). The AF70 control and genetically complemented strain produced CPA and AFT but neither of these secondary metabolites were detected in the Δ*hbx1* mutant. Due to the observed loss of aflatoxin production in the AF70 Δ*hbx1* mutant, gene expression analyses were performed on the aflatoxin pathway-specific transcription factor gene *aflR* as well as the aflatoxin biosynthetic genes *aflC* (*pksA*), *aflD* (*nor-1*), and *aflM* (*ver-1*) (Figure 8B). Significant down-regulation (≥90%) in expression of all three biosynthetic genes as well as *aflR* was observed for the Δ*hbx1* mutant at all time points compared to the control strain. Expression of these four genes was observed in the AF70 Δ*hbx1* complementation strain.

## 3. Discussion

Coordination and control of development and secondary metabolism in *A. flavus* requires the complex interaction of a number of global regulators, pathway-specific transcription factors and signaling pathways that respond to environmental cues (reviewed in [13,15,45]). Strategies for control of *A. flavus* colonization and aflatoxin contamination of food and feed crops will be dependent on identifying key genes that serve as master controllers of development and secondary metabolism in this fungus. Homeodomain proteins are well conserved in eukaryotes and are known to control development and differentiation. To our knowledge, no reports on the functional characterization of homeobox genes in aspergilli exist. In this report, we demonstrate that the *A. flavus* homeobox gene *hbx1* is required for normal vegetative growth and conidiation. We also show for the first time in a fungus that a homeobox gene, *A. flavus hbx1*, is required for sclerotial development as well as secondary metabolite production.

While significant differences in vegetative growth, conidiation and sclerotial production were noted in some of the CA14 Δ*hbx2–8* mutants compared to the control strain, total abrogation of conidial and sclerotial formation was only observed in the CA14 Δ*hbx1* mutant. As observed with the CA14 Δ*hbx1* mutant, the AF70 Δ*hbx1* mutant also did not produce conidia and sclerotia. Interestingly, while the CA14 mutant showed a slight but insignificant increase in vegetative growth as determined by colony diameter after 7 days growth, the AF70 Δ*hbx1* mutant showed a significant increase in growth compared to the control. The observed difference in colony diameter of the CA14 and AF70 Δ*hbx1* mutants compared to their respective controls may indicate that regulation of hyphal growth by *hbx1* is strain dependent and may be correlated with sclerotial morphotype. Further studies will be required to confirm this theory.

The *A. flavus hbx1* gene is an ortholog of the *M. oryzae hox2* and *Fusarium graminearum htf1* homeobox genes, both of which have been shown to be involved in conidiophore development and conidiation [40,41]. While macroconidial formation was severely reduced in *F. graminearum*, total loss of conidial production was observed in *M. oryzae hox2* mutants. As observed in our study, the defects in conidiation in the *F. graminearum* and *M. oryzae* homeobox mutants could be rescued by complementation of the mutants with a wild-type copy of the homeobox gene. Light microscopic examination of aerial hyphal structures in the Δ*hbx1* mutants indicated that there were no conidiophores produced, only septate hyphae. This was not the case in *M. oryzae* or *F. graminearum* as both produced conidiophores though in *F. graminearum* no phialides were produced [41]. Confocal laser scanning microscopy (CLSM) showed that an Hbx1::GFP fusion protein localized to nuclei of mycelia and developing conidiophores as would be expected for a transcription factor. Interestingly, CLSM indicated that no nuclei were present in the nascent vesicle (Figure 7B), suggesting that there is temporal and spatial regulation of migration of nuclei from the stipe to the developing vesicle. Active trafficking of nuclei from vesicles to phialides and then to conidia has been demonstrated in *Aspergillus oryzae* [46], but they did not report on trafficking of nuclei from stipe to vesicle. While most of the Hbx1 protein was detected in nuclei of the stipe, vesicle and the metular/phialide region of the mature conidiophore, only low levels of GFP signal were detected in conidia and even less in germinating conidia (Figure 7B). This suggests that Hbx1 may be required for conidiophore development including production of conidia but it may not be required for subsequent conidial functions such as germination. Detection of GFP fluorescence in nuclei of mycelia in a *F. graminearum* strain expressing a *htf1-gfp* fusion construct correlated with the onset of conidiogenesis; however, this study did not report if the fusion protein localized to conidia [41]. The significant decrease in expression of the conidiation-specific transcription factors *brlA* and *abaA* in the AF70 Δ*hbx1* mutant is most likely the mechanism by which conidiophore development and conidiation are disrupted. This would suggest that the *Hbx1* protein may interact with upstream regions of *brlA* and regulate its expression, which in turn would result in reduced levels of *abaA* expression, as this gene has been shown to be regulated by BrlA (reviewed in Krijgsheld et al., 2013). Similar loss of conidiophore production was noted in *brlA* mutants of *A. oryzae* [47] and *A. fumigatus* [48].

The current study also demonstrates that *A. flavus* Δ*hbx1*mutants do not form sclerotia. Recent evidence suggests that *A. flavus* sclerotia are naturally produced on the crop where they are subsequently fertilized by sexually compatible strains either on the crop or in the soil [6]. Very little is known about structural genes that are directly involved in biogenesis of sclerotia, yet a number of studies have identified global regulators in *A. flavus* that control sclerotial development [27,29,31]. While none of the previous studies on the biological roles of homeobox genes in fungi have addressed sclerotial morphogenesis, it was shown that deletion of a number of homeobox genes in the filamentous ascomycete, *P. anserina*, resulted in defects in fruiting body (perithecium) structure [38]. Although the mechanism by which *hbx1* regulates sclerotial morphogenesis is not known, we found that expression of the global regulatory factors *veA* and *nsdC*, both required for sclerotial production in *A. flavus* [29,31], were significantly reduced in the Δ*hbx1* mutant. Interestingly, expression of *sclR*, a regulatory gene of sclerotial production in *A. oryzae* [49] was not significantly impacted by *hbx1*. It is possible that loss of *hbx1* expression in the mutant may lead to dysregulation of other genetic components involved in sclerotial development that interact with Hbx1, causing loss of sclerotial production. These results demonstrate that the *hbx1* gene is required for asexual reproduction in *A. flavus*. Though not directly addressed in the current study, the role of *hbx1* in sclerotial production suggests that it would be essential for production of ascocarp-containing stromata, and therefore, also required for sexual reproduction.

The *A. flavus hbx1* mutant was unable to produce aflatoxin B_1_ and B_2_, which is consistent with the down-regulation of the aflatoxin pathway-specific transcriptional activator gene *aflR* and the aflatoxin biosynthetic genes *aflC*, *aflD* and *aflM*. It also lost the ability to produce the toxic secondary metabolites, CPA and aflatrem, indicating an expanded role for this transcription factor in regulation of *A. flavus* secondary metabolism.

In conclusion, we have shown that the *A. flavus* homeobox gene, *hbx1*, is required for normal vegetative growth and production of conidia and sclerotia. In addition, *hbx1* is also required for the biosynthesis of aflatoxins, CPA and aflatrem. This is the first report on the characterization of the biological functions of homeobox genes in an *Aspergillus* species and extends the role of fungal homeobox genes to include regulation of sclerotial and secondary metabolite production.

## 4. Materials and Methods

### 4.1. Strains, Media and Growth Conditions

*Aspergillus flavus* strains used in this study are listed in Table S1. *Aspergillus flavus* CA14 PTSΔ*pyrG* parental strain [50] (referred to as CA14 in this study; SRRC 1709) and AF70 Δ*ku70*, Δ*niaD*, Δ*pyrG* parental strain (referred to as AF70 in this study; SRRC 1713) were used as hosts for transformation. Both strains are sensitive to pyrithiamine (PT). Homeobox gene knockout mutants were generated from the CA14 or AF70 parent (Δ*hbx1* only) using pyrithiamine selection. In most cases, functional studies of the *hbx1* gene were carried out using the AF70 parent as it produced significantly higher levels of sclerotia and aflatoxins than the CA14 parent. Unless stated otherwise, the control strains for all experiments were CA14 and AF70 transformed with vector pPTRI (Takara Bio Inc., Shiga, Japan) that contains the pyrithiamine (*ptrA*) resistance gene. Cultures were point inoculated onto double strength 5/2 agar [50 mL V8 juice, 40 g agar, pH 5.2 per liter of medium [51]] supplemented with 3.0 g ammonium sulfate and 1 mg/mL uracil (2X V8 ASU) and incubated at 30 °C in the light, a condition that promotes conidiation. Conidia were collected from plates in 0.01% Triton X-100 and stored at 4 °C. Top agarose (0.5% agarose I, Amresco, Solon, OH, USA) was used on 2X V8 ASU plates for growth of Δ*hbx1* strains for real time quantitative PCR experiments. Peptone minimal salts medium [52] was used in experiments to suppress aflatoxin production. For developmental studies and promotion of aflatoxin biosynthesis, cultures were grown on potato dextrose agar (PDA, EMD, Damstatd, Germany) or Wickerham’s medium (WKM) [53] supplemented with 1 mg/mL uracil.

### 4.2. Vector Construction and Fungal Transformation

Eight identified homeobox genes were knocked out respectively in CA14 using deletion vectors constructed by the overlap fusion PCR method of Szewczyk et al. [54]. The *hbx1* gene was also knocked out in AF70. Briefly, homeobox gene-specific primer pairs were used to amplify the flanking 5′ and 3′ regions of each target homeobox gene with CA14 genomic DNA as the template and the *ptrA* gene selection marker was amplified from pPTRI. The three amplified PCR fragments were then fused sequentially in another round of PCR amplification. Lastly, nested primer pairs were used to make the final PCR product to be used for transformation. PCR amplification reactions were carried out using AccuPrime *Pfx* PCR Supermix (Invitrogen, Carlsbad, CA, USA). Tables S2 and S3 list all primers used. The AF70 Δ*hbx1* #4 mutant was genetically complemented by a PCR fragment amplified with primers *hbx1* prom-F and *hbx1* term-pyrG-R; this linear fragment contained the full-length *hbx1* gene and also included 3 kb of the 5′ UTR and 456 bp of the 3′ UTR (total size of 5654 kb). The *A. parasiticus pyrG* selection marker gene was amplified using primers pyrG-F and pyrG-R. It was fused to the 5654 bp *hbx1* PCR product by overlap fusion PCR, which generated a 7262 bp PCR product. The final *hbx1*-*pyrG* PCR product (7138 bp) used for genetic complementation was generated by amplifying the 7262 bp PCR product with nested primers *hbx1* nest-F and *hbx1* nest-R. Overlap fusion PCR was also used to generate the C-terminal GFP-tagged *hbx1* expression construct (Figure S7A). The *egfp*-*nmt1*-terminator sequence was amplified from a pUC18-based vector harboring *egfp* (Takara Bio) under the control of the *A. nidulans gpd* promoter and *A. parasiticus nmt1* transcriptional terminator region [55]. The *pyrG* selection marker was amplified from *A. parasiticus* genomic DNA with primers having overlapping sequence with the *nmt1* terminator region. The final linear fusion construct, *hbx1*-eGFP-*nmt1-pyrG*, was generated with nested primers and transformed into the CA14 Δ*hbx1*#1 mutant. Nuclear localization of Hbx1 in fungal cells was demonstrated by introduction of vector pJES35 (obtained from N. Keller, Univ. of Wisconsin, Madison, WI, USA) expressing the histone H2A-mcherry gene under the control of the *A. nidulans gpd* promoter into the CA14 *hbx1*-eGFP-*nmt1-pyrG* #18 strain. The pJES35 vector was introduced into the CA14 *hbx1*-eGFP-*nmt1-pyrG* #18 strain by co-transformation using the *A. parasiticus niaD* gene selectable marker that was PCR amplified from plasmid pSL82 [56].

Fungal protoplasts for transformation were prepared as previously described [57]. Transformants were selected on PT regeneration plates. In short, PT was added to Czepak-Dox Agar (Becton Dickinson and Company, Sparks, MA, USA) medium supplemented with 10 mM ammonium sulfate at a concentration of 0.1 μg mL^−1^ and 1 mg mL^−1^ uracil (CZAU). Regeneration plates were incubated at 30 °C for up to 7–12 days. Selected isolates were transferred to fresh CZAU plates containing PT. These isolates were used for further analysis.

### 4.3. Nucleic Acid Analysis

Fungal genomic DNA for PCR was isolated using the MasterPure Yeast DNA Purification Kit (Epicentre, Madison, WI, USA) according to the manufacturer’s instructions. To confirm the successful integration of either knockout, complementation or GFP fusion cassettes, transformant genomic DNA was amplified with ExTaq HS polymerase (Takara Bio, Inc., Kusatsu, Japan) using specific primer pairs listed in Tables S1 and S2.

### 4.4. Quantitative PCR Analysis

For real time quantitative PCR (RT-qPCR) experiments, conidial suspensions for growth medium inoculum were collected by gentle scraping the colony surface with a 10 mL of 0.01% Triton X-100. Due to their inability to produce conidia, a small piece of mycelia from the CA14 Δ*hbx1 #*1 and AF70 Δ*hbx1* #4 mutants was center point inoculated of the onto 2X V8 ASU top agarose (0.5% agarose I, Amresco, Solon, OH, USA) plates. Following growth for 7 d in the light at 30 °C, the top agarose layer containing the Δ*hbx1* (ΔniaD, ΔpyrG, ptrA+) mycelia was collected and macerated in 30 mL peptone minimal salts supplemented with uracil (PMSU—not conducive to aflatoxin production) using a tissue grinder (Tissumizer SDT1810, Tekmar, Cincinnati, OH, USA). Thirty mL of the macerated AF70 Δ*hbx1* #4 strain and about 5 × 10^5^ conidia/mL of the control (AF70 ΔniaD, pyrG+, ptrA+) and genetically complemented strain (AF70 Δ*hbx1*-com #8; ΔniaD, pyrG+, ptrA+) were used to inoculate 500 mL of PMSU broth in a 1 L Ehrlenmeyer flask. Cultures were incubated for 24 h at 30 °C in the dark with agitation at 280 rpm. The mycelia were then collected under vacuum on sterile miracloth, and approximately 0.5 g wet weight of each strain was transferred into 25 mL PDBU (conducive to conidial, sclerotial and aflatoxin production) in a 250 mL Ehrlenmeyer flask. Four flasks of each strain were inoculated to serve as samples for RT-qPCR analysis. Cultures were placed in the dark with static incubation at 30 °C for 6, 24 and 48 h. Mycelia for RT-qPCR analysis were collected for each time point on sterile miracloth and immediately frozen in liquid nitrogen and stored at −80 °C. RNA was isolated from about 100–200 mg of ground mycelia using the TRI Reagent with the Direct-zol RNA MiniPrep kit (ZYMO Research, Irvine, CA, USA). RNA quality and quantity were determined using the Experion Automated Electrophoresis Station (Bio-Rad). Aliquots (2 μg) of RNA from each sample were treated in 20 μL reactions using the protocol for the Turbo DNA-free kit (Thermo Fisher Scientific). The DNase treated RNA was diluted using nuclease free water to a working concentration of 20–40 ng/μL and stored at −80 °C. A one step RT-PCR procedure was performed using the Power SYBR Green RNA to Ct 1-Step Kit (Applied Biosystems) in the 48 well StepOne Plus instrument (StepOne software version 2.0, Applied Biosystems) using 10 μL final volume reactions for each sample, done in triplicate. The relative gene expression levels for all time points were normalized to the *A. flavus* 18S rRNA C_T_ values as part of the ΔΔC_T_ analysis method. A Fold-Change formula (2^−ΔΔC^_T_) [44] was used in the conversion of the ΔΔC_T_ results for a final calculation of values. Primers used for RT-qPCR are listed in Table S4.

### 4.5. Analysis of Growth and Development

A 1 μL aliquot of conidial suspension (~3 × 10^5^ conidia/mL) of the CA14 Δ*hbx* mutants (except Δ*hbx1* as it did not conidiate) and control strains was center-point inoculated onto a small Petri plate (Falcon; 60 × 15 mm) of PDAU and WKMU agar (a medium strongly-conducive to sclerotial production) for analysis of conidial and sclerotial production. Cultures were incubated in the light at 30 °C for 7 days for analysis of conidial production. For sclerotial production, CA14 strains were inoculated onto WKMU plates and incubated for 14 days in the dark at 30 °C. Inoculations were performed in triplicate. For growth of the AF70 control, Δ*hbx1* #4 mutant and Δ*hbx1*-com #8 strain, a 5 μL aliquot of macerated hyphal fragments was center-point inoculated onto a Petri plate (Falcon; 100 × 15 mm). Colony growth was determined by measuring colony diameter (6 replicates) after seven days on WKMU under white light or in the dark at 30 °C. Conidial and sclerotial production (6 replicates) was quantified after growth for seven (conidia) or 14 days (sclerotia) on WKMU agar under white light or in the dark at 30 °C. Following incubation for 7 days, agar plates were flooded with 10 mL of sterile 0.01% Triton X-100 solution and conidia gently scraped from the surface of the plate then transferred to a sterile 15 mL Sarstedt tube. When necessary, dilutions of conidial suspensions were made in 0.01% Triton X-100 and conidia counted using a hemocytometer (SPotlite hemocytometer, McGaw Park, IL, USA). For determination of sclerotial numbers, a single 6 mm plug was removed from a point on the colony that was half the distance from the center to the edge of the colony. Sclerotia were teased off of the agar plug, collected on the surface of a piece of filter paper and counted. Statistical analysis was carried out using one-way ANOVA followed by Dunnett’s Multiple Comparison Test (GraphPad Prism 5, La Jolla, CA, USA). Values of bars with asterisks are significantly different by analysis of variance (*p* < 0.05).

### 4.6. Analysis of Secondary Metabolite Production in Δhbx1 Mutants

For aflatoxin analysis, strains were grown on WKMU agar for 7 days in the dark at 30 °C. The mycelia and agar were collected, lyophilized, dry weight determined and extracted for analysis of aflatoxin production. The lyophilized material was extracted with ethyl acetate/acetone (1:1)/0.1% formic acid (20 mL) for 24 h at room temperature. The extract was filtered, and the filtrate was concentrated under nitrogen to dryness. Each extract was redissolved in acetonitrile (~1 mg/mL), filtered through a Spin-X centrifuge spin tube filter (Costar), then analyzed on a Waters Acquity UPLC system (40% MeOH in water, BEH C18 1.7 µm, 2.1 × 50 mm column) using fluorescence detection (ex = 365 nm, em = 440 nm). Cultures for CPA and aflatrem (AFT) analysis were grown on WKMU agar at 30 °C in the dark for 14 days. The agar was collected, lyophilized and extracted with ethyl acetate/0.1% formic acid for 24 h at room temperature (2×). The extracts were concentrated *in vacuo*. The dried extracts were redissolved in methanol at 5 mg/mL and filtered for analysis on the Waters Acquity UPLC system using PDA detection and the following gradient solvent system (solvent A: 0.1% formic acid in water; solvent B: 0.1% formic acid in acetonitrile): 5% B (0–2.5 min), gradient to 25% B (2.5–3 min), gradient to 100% B (3–10 min), 100% B (10–15 min), then column equilibration 5% B (15.1–20.1 min). Cyclopiazonic acid standard was used to confirm identity (CPA, rt = 4.10 min), while AFT was identified based on UV spectra (λ_max_ 231.1, 282.6, 292.0 (sh) nm.), which corresponded to literature values [58]. Aflatoxin B_1_, B_2_ and CPA standards were purchased from Sigma Aldrich (St. Louis, MO, USA). Aflatrem was a kind gift from Jim Gloer (U. Iowa). Three biological replicates of each culture were collected for analysis of secondary metabolites.

### 4.7. Phylogenetic Analysis

A neighbor-joining tree was constructed using CLC Sequence Viewer software (Qiagen, Germantown, MD, USA) based on the deduced amino acid sequences of representative fungal homeodomain transcription factors identified using the InterPro homeodomain term (IPR001356). The graphics showing the location of the InterPro domains were created using the Comparative Fungal Genomics Platform’s (http://cfgp.riceblast.snu.ac.kr) InterPro Scan tool (version 58).

### 4.8. Microscopy

For imaging procedures, fungi were inoculated onto 2X V8 ASU plates and incubated in the light for 7 days at 30 °C. To evaluate the overall conidiophore and/or aerial hyphal structures, the samples were visualized using a Nikon SMZ25 stereoscope (Nikon, Melville, NY, USA) equipped with an ANDOR Zyla 5.5 camera (Andor Technology Ltd., Belfast, UK). Imaging was conducted at ×120 magnification. To differentiate aerial hyphae from conidiophore stipes we performed further local analysis of the structures using a Nikon E600 microscope equipped with a Nikon DS-Qi1Mc camera at ×200 magnification.

Samples for confocal laser scanning microscopy (CLSM) were prepared on a glass-bottomed fluorodish (World Precision Instruments, Sarasota, FL, USA). The CA14 strains expressing Hbx1::GFP only or co-expressing Hbx1::GFP and the H2A::mCherry chimerae were inoculated on PDA plates and incubated at 30 °C for 16 h. The newly growing colonies were excised from the PDA plates, inverted on the fluorodish and grown for 48 h prior to fixation and imaging. In preparation for imaging, hyphae growing on the glass were fixed with 4% formaldehyde solution for 30 min, washed three times with 1X PBS buffer and mounting medium added. For the strain expressing Hbx1::GFP only, the DAPI nuclear stain was used to visualize nuclei. Prior to DAPI staining the fixed samples were permeabilized with 0.5% Triton X-100. Images were captured with confocal laser microscope (Leica model TCS SP5; Leica Microsystems CMS GmbH, Mannheim, Germany) using a 20× dry objective for overall global examination of Hbx1 localization in mycelia and 40× oil objective plus a 2× electronic zoom for conidiophore imaging. A 488 nm laser line was used to excite the GFP, while the fluorescent emission was detected from 500 to 540 nm and a 561 nm laser line was used to excite mCherry, while fluorescence emission was detected from 630 to 660 nm. DAPI stains were visualized using 405 nm laser line. Co-localization of GFP and mCherry was examined using Fiji software (Fiji, ImageJ, Wayne Rasband National Institutes of Health).

## Supplementary Materials

The following are available online at www.mdpi.com/2072-6651/9/10/315/s1, Table S1: Strains and plasmids used in this study; Table S2: Oligonucleotide primers used for construction of *hbx* gene knockout PCR products; Table S3: Oligonucleotide primers used in construction of the *hbx1*-com-pyrG and *hbx1*-GFP-nmt1-pyrG PCR products; Table S4: Oligonucleotide primers used for RT-qPCR; Figure S1: Alignment of the eight *A. flavus hbx* gene homeodomains; Figure S2: PCR confirmation of knockout of *A. flavus* CA14 *hbx* genes; Figure S3: RT-qPCR analysis of *hbx* gene expression in control and knockout transformants; Figure S4: PCR and RT-qPCR confirmation of knockout of *A. flavus* AF70 *hbx1* gene; Figure S5: Analysis of three putative AF70 Δ*hbx1*-com transformants; Figure S6: Analysis of secondary metabolites in *A. flavus* strains; Figure S7: Schematic representation and PCR confirmation of CA14 transformants carrying the *hbx1*-GFP-*nmt1* term-pyrG vector and *gpd* promoter-H2A-mCherry co-transformation.
