# Supplementary material for: The Aspergillus flavus Homeobox Gene, hbx1, Is Required for Development and Aflatoxin Production
Source: Toxins (Basel). 2017 Oct 12;9(10):315. doi: 10.3390/toxins9100315 (PMC5666362; doi:10.3390/toxins9100315)
Supplement: Supplementary file 1 [file toxins-09-00315-s001.pdf]

Supplementary Materials: The *Aspergillus flavus* homeobox gene, *hbx1*, is required for development and aflatoxin production

Jeffrey W. Cary, Pamela Harris-Coward, Leslie Scharfenstein, Brian M. Mack, Perng-Kuang Chang, Qijian Wei, Matthew Lebar, Carol Carter-Wientjes, Rajtilak Majumdar, Chandrani Mitra, Sourav Banerjee and Anindya Chanda

Table S1. Strains and plasmids used in this study.

**Strains**	**Genotype**	**Reference**
Aspergillus flavus		
CA14 SRRC 1709	*Δku70, ΔniaD, ΔpyrG, ptrA^S*	Chang et al., 2010
CA14 pPTRI	*Δku70, ΔniaD, ΔpyrG, ptrA^R*	Cary et al., 2015
AF70 SRRC 1713	*Δku70, ΔniaD, ΔpyrG, ptrA^S*	This study
AF70 pPTRI	*Δku70, ΔniaD, ΔpyrG, ptrA^R*	This study
AF70 pyrG-1	*Δku70, ΔniaD, pyrG, ptrA^S*	This study
CA14 hbx mutants	*Δku70, ΔniaD, ΔpyrG, Δhbx::ptrA*	This study
AF70 Δhbx1 #4	*Δku70, ΔniaD, ΔpyrG, Δhbx1::ptrA*	This study
AF70 Δhbx1-com #8	*Δku70, ΔniaD, Δhbx1::ptrA, hbx1::pyrG*	This study
CA14 hbx1-GFP-nmt1-pyrG #18	*Δku70, ΔniaD, gpdA(p)::hbx1::egfp::nmt1(t)::pyrG*	This study
CA14 hbx1-GFP-H2A-mcherry	*Δku70, gpdA(p)::hbx1::egfp::nmt1(t)::pyrG, H2A::mcherry, niaD*	This study
Plasmids		
pPTRI	*Aspergillus oryzae ptrA*	Cary et al., 2015
pUC18-gpd-GFP-nmt1	*gpd(p)::egfp::nmt1(t)*	Rajasekaran et al., 2008
pJES42.1	*gpdA(p)::H2A::mcherry*	Spraker and Keller [a]
pSL82	*Aspergillus parasiticus niaD*	Horng et al., 1990

[a] unpublished data

Table S2. Oligonucleotide primers used for construction of *hbx* gene knockout PCR products

**Primer designation**	**Oligonucleotide sequence (5′-3′)**
Fusion PCR knockout	
ptrA-F	GGGCAATTGATTACGGG
ptrA-R	TGACGATGAGCCGCTCTTGC
hbx1 F1-F	CCAATCTCTCCATCCGCTTGT
hbx1 F1-R	gggatcccgtaatcaattgcccTCCAGGGTCTCAACTTGT
hbx1 F3-F	caagagcggctcatcgtcacccACACCTGCTTCTAACTCACGA
hbx1 F3-R	CAGACGCCGAAGAAACACAC
hbx1 nest-F	GGTGTCAATCCTCGTCTGGAA
hbx1 nest-R	CGGCAAAGGAAGACAGGTTC
hbx2 F1-F	TGTCTCCATCGTCATCGTGTG
hbx2 F1-R	gggatcccgtaatcaattgcccTGTCTGCTCGGAATCGCTATG
hbx2 F3-F	caagagcggctcatcgtcacccTCCCAACGCCAATCTCCCTT
hbx2 F3-R	GGTGTCCTTCCTCATCTGTCT
hbx2 nest-F	CATCCATCCTTCGTTCGTCTC
hbx2 nest-R	ACAAGCAAACCGTGGTAATGC
hbx3 F1-F	ATAACCGAGACCACCAAGCC
hbx3 F1-R	gggatcccgtaatcaattgcccGGATTGTTTGATGGCGGGTT
hbx3 F3-F	caagagcggctcatcgtcacccTGGTAAAGTTCGTCCCGCTC
hbx3 F3-R	TGGCTGAAAGGATGACGACG
hbx3 nest-F	CCCAAGACCAAGGACAGTTAC
hbx3 nest-R	AAGGTGGTCGTTGTCAGGATT
hbx4 F1-F	CCAGGATTACCACCAACGCA
hbx4 F1-R	gggatcccgtaatcaattgcccATCGCAGCCCTCACCAGTTT
hbx4 F3-F	caagagcggctcatcgtcacccTGGCATACAACAACTTCGCTG
hbx4 F3-R	CAACCCTAATCCCGCCAAC
hbx4 nest-F	TCCTCCACCCGTTTGTCTCT
hbx4 nest-R	CGGCATTGGAAAGAAGCGAAG
hbx5 F1-F	GATGCTGGTTGGTTCCTTCGT
hbx5 F1-R	gggatcccgtaatcaattgcccAGAGTCGCCGTTGATAGGAAA
hbx5 F3-F	caagagcggctcatcgtcacccTCTGAAACCAACCATCTCCCA
hbx5 F3-R	CACAAACAAGGAAGAGCAGCA
hbx5 nest-F	GCACAGCAAAGAGGAATGGGTC
hbx5 nest-R	CACCGCATTCCTCAAGACAC
hbx6 F1-F	GTTCCCTGGTGGAGAAGTCG
hbx6 F1-R	gggatcccgtaatcaattgcccTCGTCAATTGAGGCATGGCT
hbx6 F3-F	caagagcggctcatcgtcacccTGGAACAGTGGCGAGATTCC
hbx6 F3-R	GGAGCTGGTCAAGGAGATCG
hbx6 nest-F	ATTGTCCTTGCTCGGGATCG
hbx6 nest-R	AAGGTGGGGAGTTCGTCAGT
hbx7 F1-F	CTTTCCCAAACCAGACGCAC
hbx7 F1-R	gggatcccgtaatcaattgcccTCTGCCGTGCGAGTAACTT
hbx7 F3-F	caagagcggctcatcgtcacccGTAGCAACATCAGAAGCACCA
hbx7 F3-R	GCTCGCAACTTGGACATAACT
hbx7 nest-F	CCGTTGGTATGGGTGTAGTTG
hbx7 nest-R	CGTTTGCTTTACGCTGCGAC
hbx8 F1-F	GTGCCCGTTGTTGCATTCTT
hbx8 F1-R	gggatcccgtaatcaattgcccAACTCTCTTGGGGCTTGGGG
hbx8 F3-F	caagagcggctcatcgtcacccGGTATTCTCGACCAGGCGAG
hbx8 F3-R	GCGCAGAGGTGAAAAGCAAA
hbx8 nest-F	GCCGTCGTTCAGGACTTCTT
hbx8 nest-R	CACAACTGCTAGCGAGTCCT

lower case indicates overlap with either the ptrA-F or ptrA-R selectable marker sequence

Table S3. Oligonucleotide primers used in construction of the *hbx1*-com-pyrG and *hbx1*-GFP-nmt1-pyrG PCR products.

Primer designation	Oligonucleotide sequence (5′-3′)
Fusion Δ*hbx1*-com construct	
hbx1 prom-F	CGGCTAACCAGAGGGAATCC
hbx1 term-R	tatagaagcacttaccttcgcatTAATGAGTGGTGCAGCACGT
pyrG-F	ATGCGAAGGTAAGTGCTTCT
pyrG-R	TGAAACATGACCCTTGACTCTGA
hbx1 nest-F	TGGGCAGGTTTCACAGATGG
pyrG nest-R	GTCCATATCTCGAGGCAGGC
Fusion *hbx1*-GFP construct	CGGCTAACCAGAGGGAATCC
hbx1 prom-F	acagctcctcgcccttgctcacATTTCCTCTCCAATCGCTCGG
hbx1 GFP-R	GTGAGCAAGGGCGAGGAG
GFP-F	GGATGATGTTGAGTTCGCGC
nmt1-R	cagcgcgaactcaacatcatccATGCGAAGGTAAGTGCTTCT
nmt1 pyrG-F	TGAAACATGACCCTTGACTCTGA
pyrG-R	TGGGCAGGTTTCACAGATGG
hbx1 nest-F	GTCCATATCTCGAGGCAGGC
pyrG nest-R	

[a] lower case sequence overlaps with pyrG-F selectable marker sequence

[b] lower case sequence overlaps with GFP-F reporter gene sequence

[c] lower case sequence overlaps with nmt1-R terminator sequence

Table S4. Oligonucleotide primers used for qRT-PCR

**Primer designation**	**Oligonucleotide sequence (5′-3′)**
hbx1-F	TCGGGTTGCTAACTGGTTCCA
hbx1-R	CTCTTCTTTGACGTCTGGGTT
hbx2-F	CCGATCGACTCCAAGAACAAA
hbx2-R	CTAGGATAAGGGTGGTCCAGAT
hbx3-F	GCCGTGGATAATGGTGTCTAT
hbx3-R	CGGTTGGGATGTAGGTATTCTC
hbx4-F	CGTTGGAGTCGTTCCAGAAT
hbx4-R	AAGCAGGGCGTTGGATAAA
hbx5-F	CGCCGGAAACCCAATTAAAC
hbx5-R	CAGGGATGTGGAGGACAATAAG
hbx6-F	GCACACCACGCCGATAATTC
hbx6-R	TTGCTCGACACAGCATTCCT
hbx7-F	TGACTCTTTCACGTCGGATATG
hbx7-R	CTCTTGCGTCCCTTCCTTATT
hbx8-F	ACCGGTTCCGTTTCCCTTTT
hbx8-R	GTTGAAGTCCGTCCGTCAGT
aflR-F	CTCAAGGTGCTGGCATGGTA
aflR-R	CAGCTGCCACTGTTGGTTTC
aflC-F	CGCCACCTATTTTGCCGATG
aflC-R	GTACTCAGACACAGACCGGC
aflD-F	CAGCACCATCACCAACATGC
aflD-R	CTGCACATGTCCTGGATCGA
aflM-F	CGCCACCTATTTTGCCGATG
aflM-R	GTACTCAGACACAGACCGGC
brlA-F	CGCTTATGATGACAACGTGGA
brlA-R	GAACCATAGGAGGGCATTG
abaA-F	GCTTTCCAGCAAGAGCCCTTG
abaA-R	ACCTGCTTTCTCGACCTCCTT
veA-F	TCATCGTAGTCGTAGTCATC
veA-R	GTTTCGTCTCGAGCGCCA
nsdC-F	ACAGCACGTCCCTTATGAATAC
nsdC-R	GCAAGTCCATTTCATCCCTTTG
18S-F	TTCCTAGCGAGCCCAACCT
18S-R	CCCGCCGAAGCAACTAAG

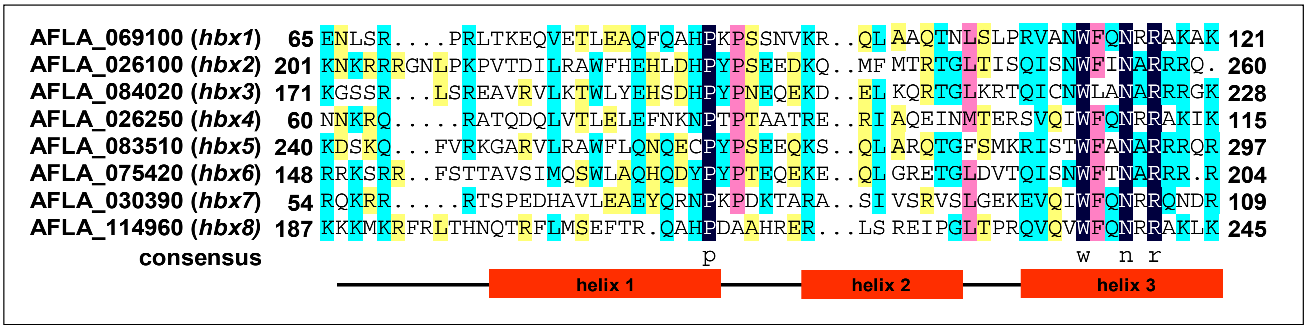

Figure S1. Alignment of the eight *A. flavus hbx* gene homeodomains. The alignment was obtained using ClustalW program of DNAMAN and colored according to the ClustalX color definitions. The three helical regions characteristic of the homoedomain were identified using the Chou-Fasman Secondary Structure Prediction Server (http://www.biogem.org/tool/chou-fasman/). Numbers flanking the amino acid sequences indicate the position of the first and last amino acids that comprise the deduced homeodomain.

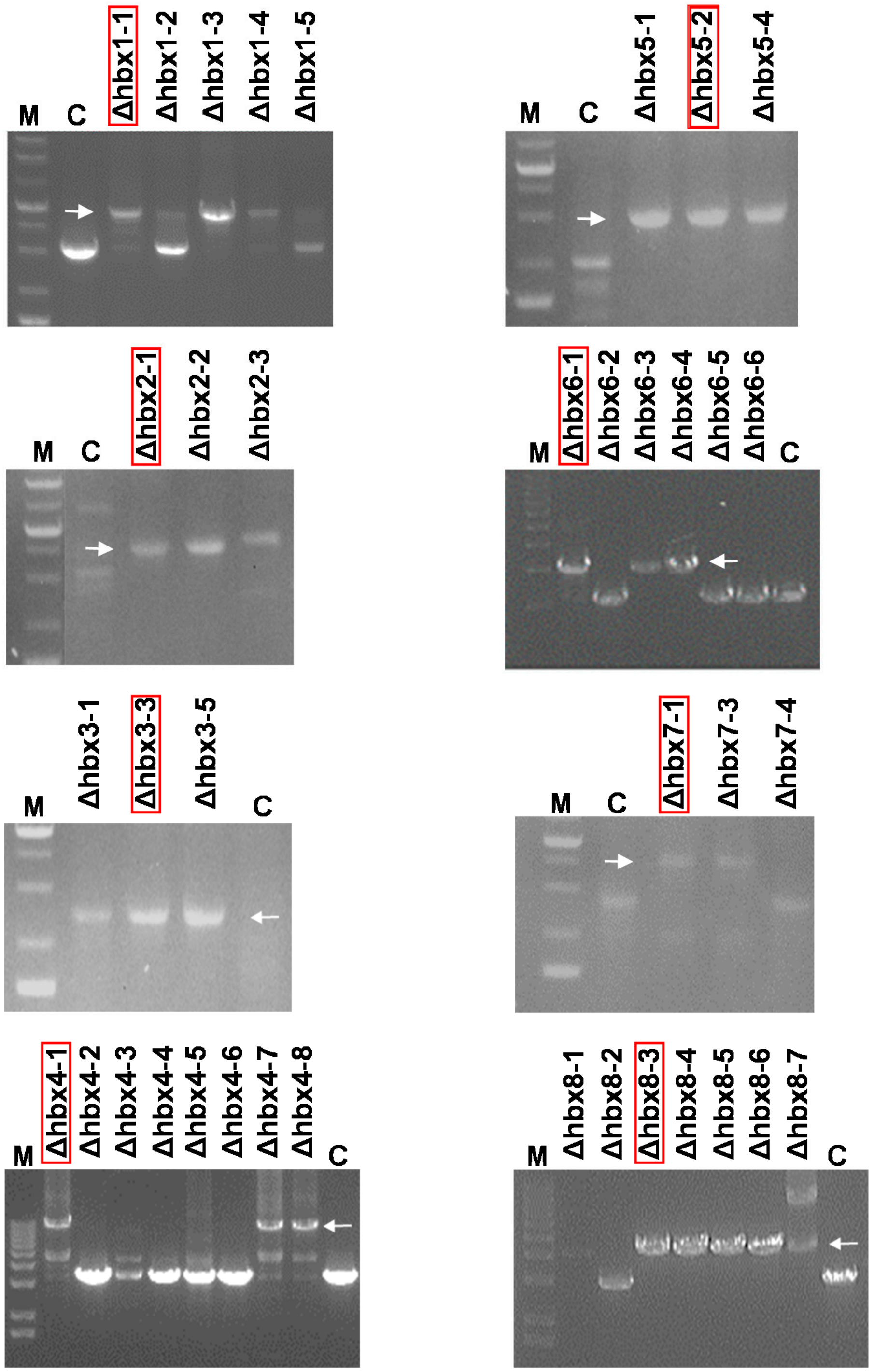

Figure S2. PCR confirmation of knockout of *A. flavus* CA14 *hbx* genes. DNA from putative *hbx* knockout transformants was amplified with primers *hbx* F1-F and *hbx* F3-R for the representative gene being analyzed. Genomic DNA was amplified with EmeraldAmp Max PCR Master Mix (Takara Bio). The PCR products were separated on a 1% agarose gel and observed following ethidium bromide staining. Gel images were captured on a Fujifilm LAS-3000 luminescent image analyzer. Arrows denote PCR product confirming successful replacement of the *hbx* gene region with the pyrithiamine (*ptrA*) selectable marker. Transformants used in subsequent experiments are boxed in red. Abbreviations: M, molecular size marker; C, CA14 control

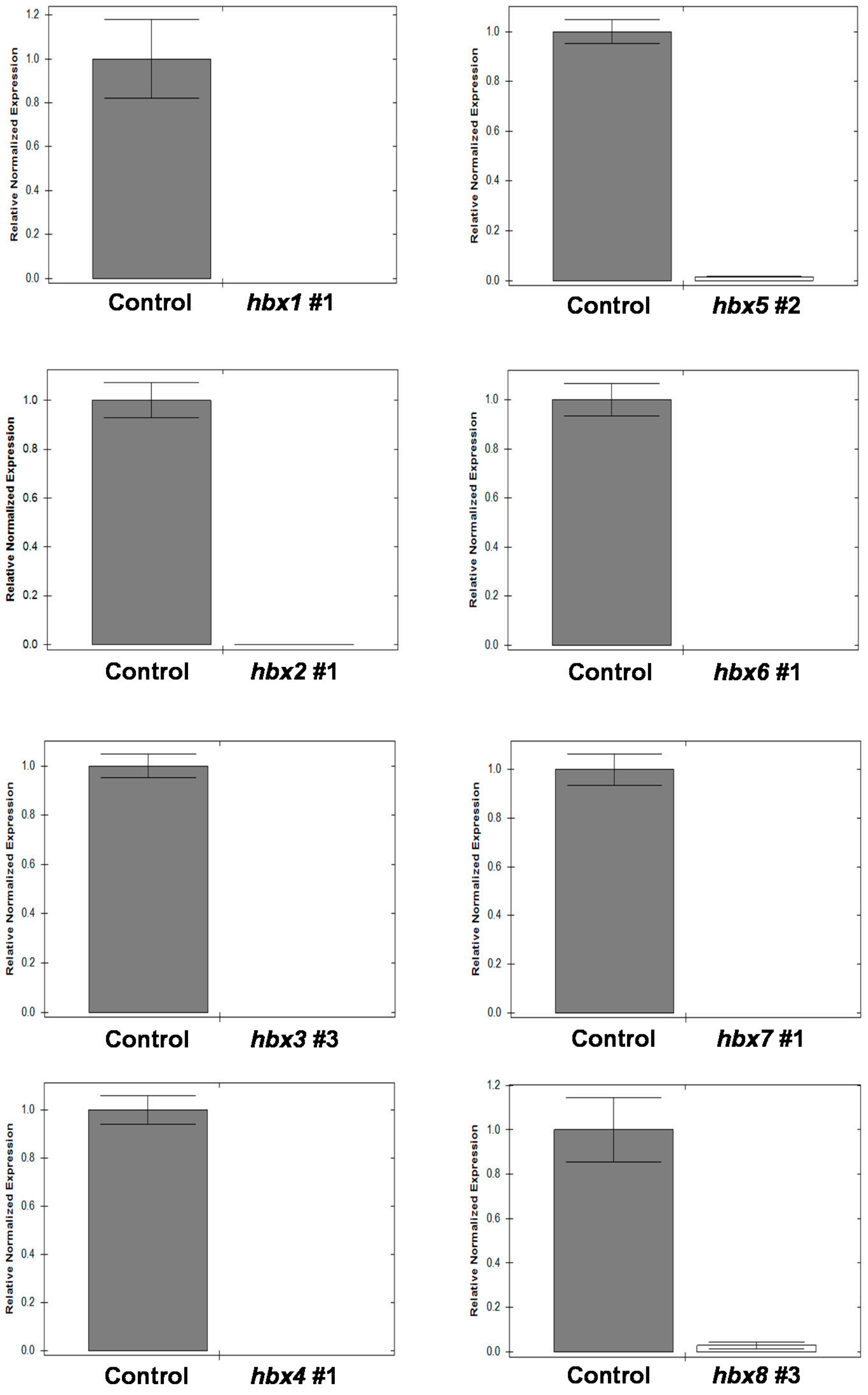

Figure S3. RT-qPCR analysis of *hbx* gene expression in control and knockout transformants. RNA was isolated from CA14 control and Δ*hbx* mutants after 48 h growth on PDAU in the dark at 30 °C. Expression levels of *hbx* genes in the Δ*hbx* mutants are relative to a level of 1 set for the corresponding control *hbx* gene. The relative gene expression levels for all time points were normalized to the *A. flavus* 18S rRNA Cq values.

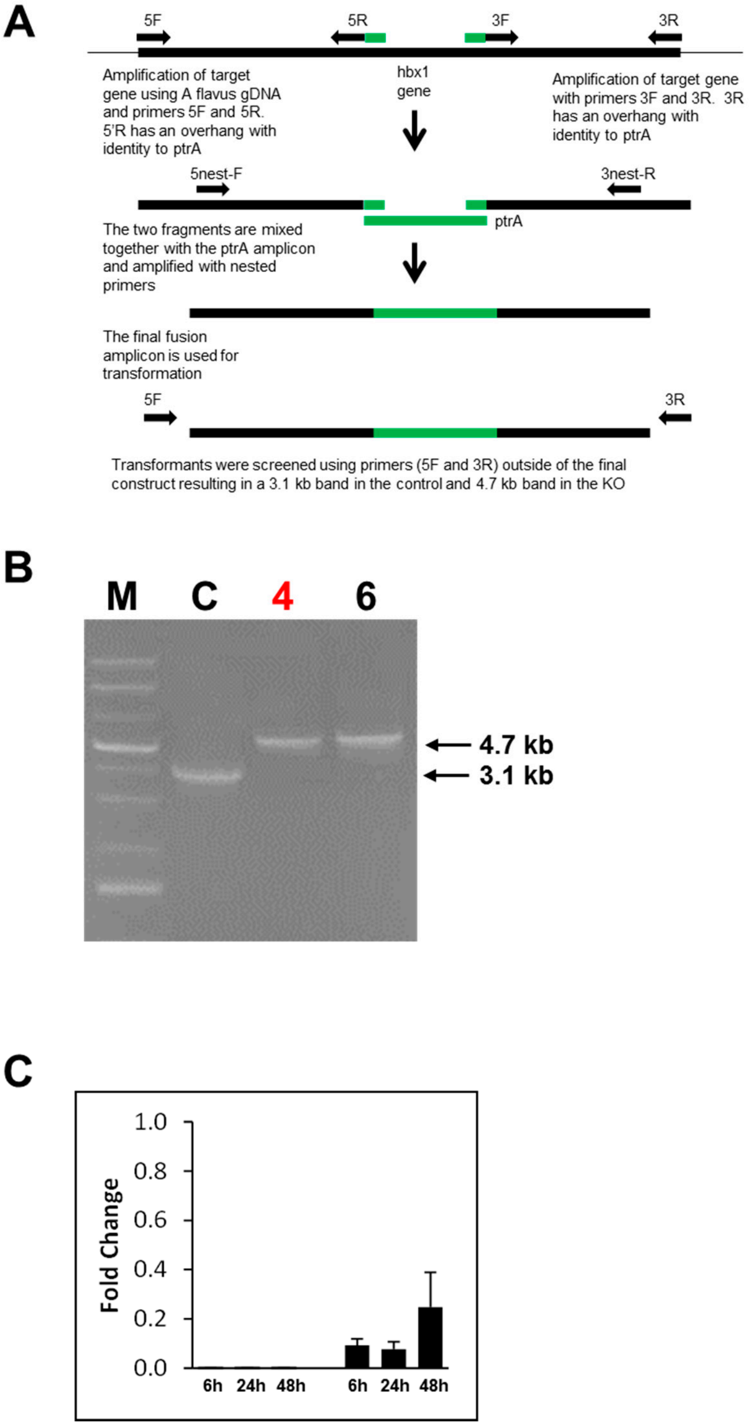

Figure S4. PCR and RT-qPCR confirmation of knockout of *A. flavus* AF70 *hbx1* gene. (**A**) Schematic of steps and primers used to confirm knock out the *hbx1* gene. (**B**) PCR confirmation of successful knockout of *hbx1* in AF70. DNA from putative *hbx1* knockout transformants was amplified with primers 5F and 3R. Genomic DNA was amplified with EmeraldAmp Max PCR Master Mix (Takara Bio). The PCR products were separated on a 1% agarose gel and observed following ethidium bromide staining. Arrows denote 4.7 kb PCR product confirming successful replacement of the *hbx1* gene region with the pyrithiamine (*ptrA*) selectable marker. Transformant used in subsequent experiments is denoted in red. Abbreviations: M, molecular size marker; C, AF70 control. (**C**) RT-qPCR analysis of *hbx1* expression in the *Δhbx1* #4 mutant (white bars) and genetically complemented *Δhbx1* #4 strain (black bars). Note total loss of *hbx1* expression in the mutant and restoration of expression in the genetically complemented *Δhbx1*-com #8 strain.

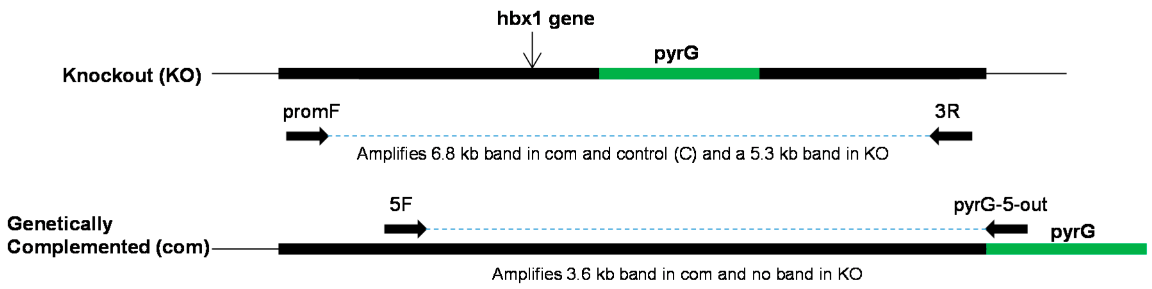

B

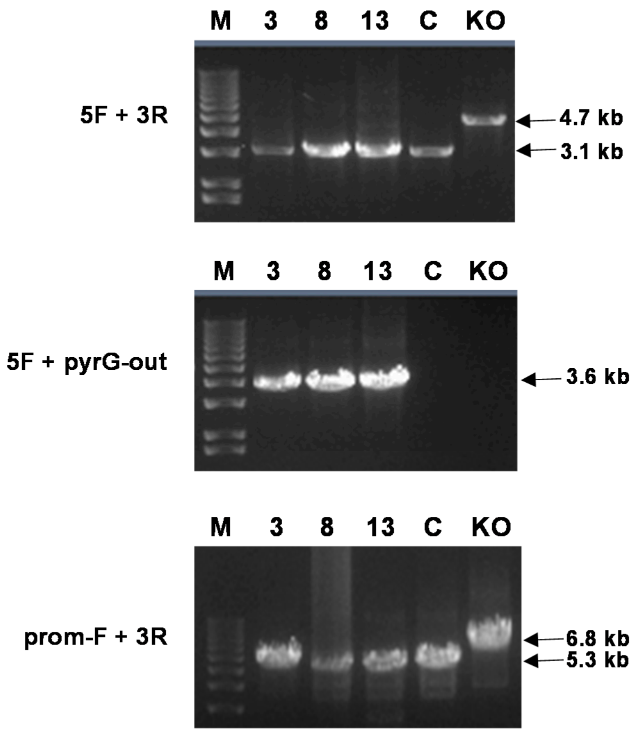

Primer pair	Com	C	KO
5F + 3R	3.1	3.1	4.7
5F + pyrG-out	3.6	None	None
prom-F + 3R	5.3	5.3	6.8

Figure S5. Analysis of three putative AF70 Δ*hbx1*-com transformants. (**A**) Schematic depicting integration of the *hbx1-pyrG* PCR product at the site of the *hbx1* deletion via homologous recombination and predicted sizes of PCR products using 3 sets of diagnostic primer pairs. (**B**) PCR confirmation that a wild-type copy of the *hbx1* gene had integrated at the site of the deleted *hbx1* gene using 3 primer pairs (5F/3R, 5F/pyrG-out, and prom-F/3R).

A

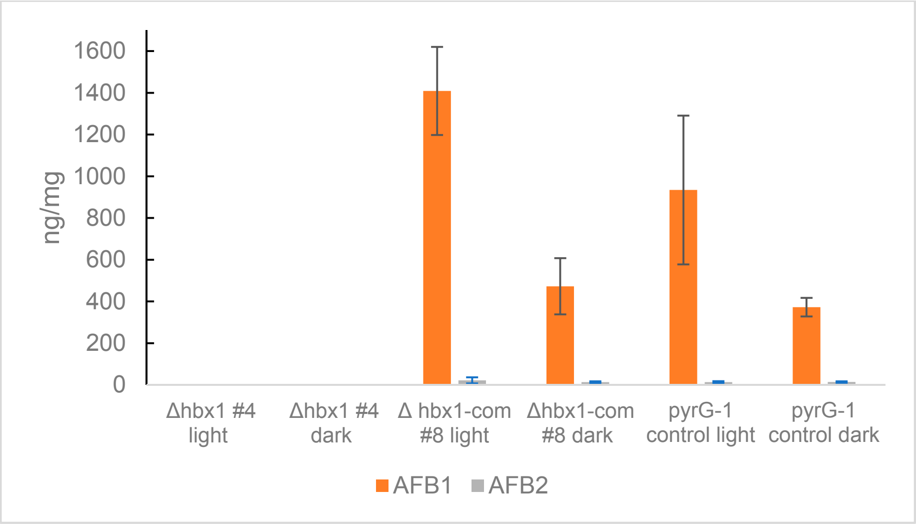

B

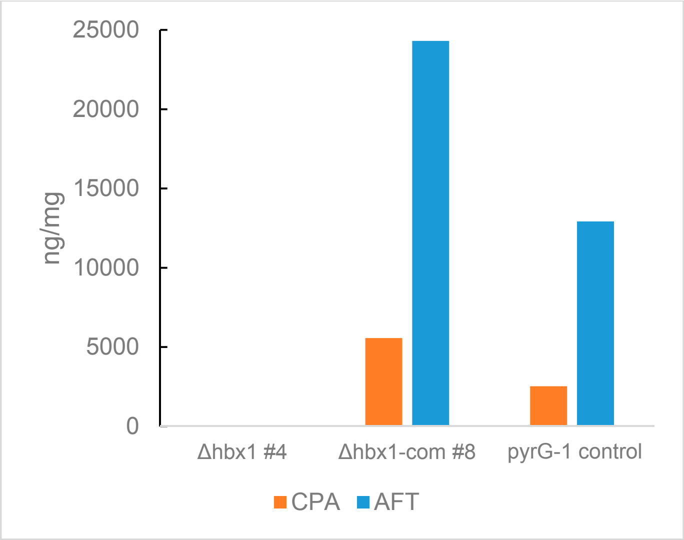

Figure S6. Analysis of secondary metabolites in *A. flavus* strains. (**A**) Aflatoxin B1 and B2 production in the AF70 control, *Δhbx1* #4 mutant and *Δhbx1*-com #8 genetically complemented strain. Strains were grown on WKMU agar for 7 days at 30 °C in the dark or with illumination. Extracts were analyzed on a Waters Acquity UPLC system using fluorescence detection (ex = 365 nm, em = 440 nm). (**B**) CPA and aflatrem (AFT) production in the A. flavus strains following growth on WKMU agar for 14 days at 30 °C in the dark or with illumination. Peak area at l = 280 nm was used to quantify both compounds from UPLC-PDA chromatograms.

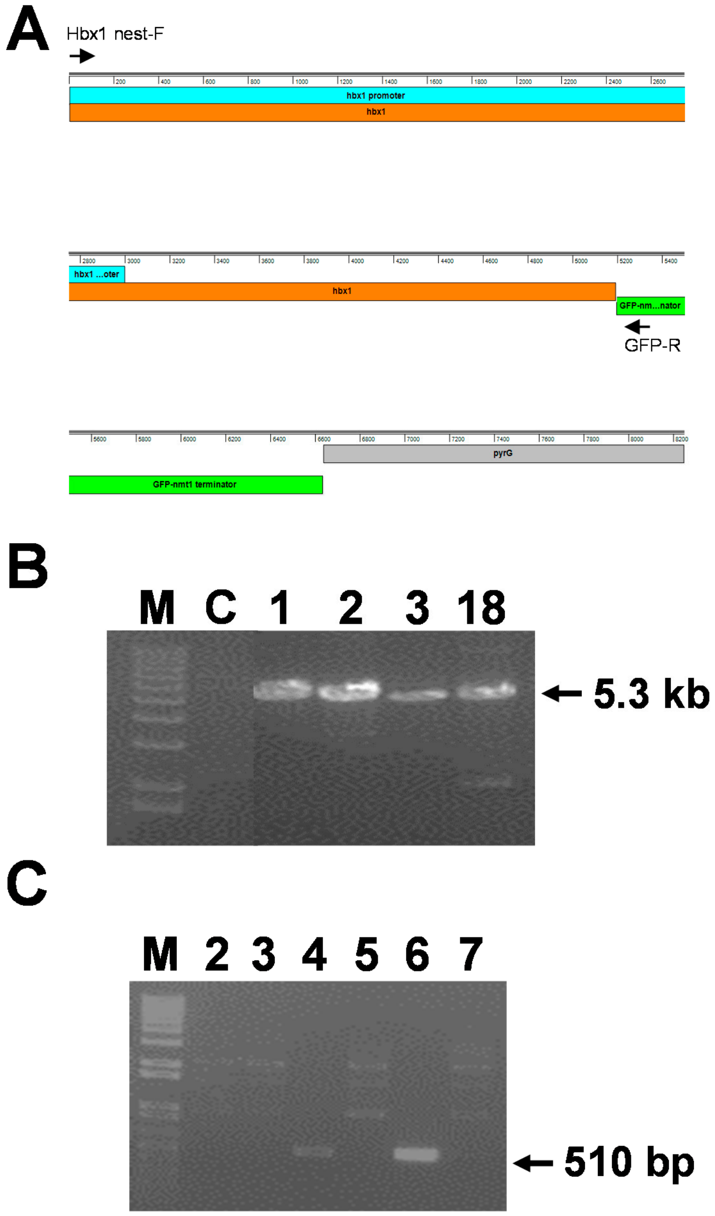

Figure S7. Schematic representation and PCR confirmation of CA14 transformants carrying the *hbx1*-GFP-*nmt1* term-pyrG vector and *gpd* promoter-H2A-mCherry co-transformation. (**A**) Schematic of the *hbx1*-GFP-*nmt1* term-pyrG PCR product used to transform CA14 for subsequent localization of the *hbx1*-GFP protein in *A. flavus* cells. (**B**) PCR confirmation of CA14 transformants harboring an integrated copy of the *hbx1*-GFP-*nmt1* term-pyrG PCR product using primers *hbx1* nest-F and GFP-R primers. A product of 5.3 kb in size was observed in all transformants but not from control DNA, as expected. Transformant #18 was used in all experiments. (**C**) PCR confirmation of CA14 co-transformants harboring an integrated copy of the *hbx1*-GFP-*nmt1* term-pyrG PCR and the *gpd*-H2A-mCherry vector. A product of the expected size of 510 bp was detected in transformants 4 and 6 following amplification of genomic DNA with primers specific for the mCherry gene region (mCherry-F, AAGCTGAAGG TCACCAAGGG and mCherry-R GTACTGCTCGACGATGGTGT). Abbreviations: M, molecular size marker; C, CA14 control.
